# Comparing Apotel and Remifentanil for Multimodal Patient-Controlled Analgesia in Postoperative Pain Management Following Total Knee Arthroplasty Surgery: A Randomized Controlled Trial

**DOI:** 10.5812/aapm-141975

**Published:** 2024-03-18

**Authors:** Seyed Ali Golrokh Moghadam, Amin Tajerian, Behnam Mahmoudieh, Mohsen Parsi Khamene, Alireza Kamali

**Affiliations:** 1 School of Medicine, Arak University of Medical Sciences, Arak, Iran; 2 Department of Anesthesiology and Critical Care, Arak University of Medical Sciences, Arak, Iran; 3 Department of Orthopedic Surgery, Arak University of Medical Sciences, Arak, Iran

**Keywords:** Knee, Arthroplasty, Pain Management, Remifentanil, Opioid Tolerance, Infusion Pumps

## Abstract

**Background:**

Total knee arthroplasty (TKA) is a standard surgical procedure for individuals with debilitating knee arthritis. Effective postoperative pain management is essential for successful recovery, although traditional opioid-based methods have limitations.

**Objectives:**

This study aimed to compare the efficacy of Apotel and remifentanil patient-controlled analgesia in managing postoperative pain after TKA.

**Methods:**

This double-blind, randomized, controlled clinical trial took place at Amir-al-Momenin and Qods Hospitals in Arak, Iran, spanning from June 2022 to September 2023. Sixty-two eligible patients scheduled for knee joint replacement were randomly assigned to receive either Apotel (Group A) or remifentanil (Group R) as part of multimodal analgesia administered via a pain pump for postoperative pain relief in TKA. The study assessed hemodynamic parameters, pain levels (measured using the Visual Analog Scale), analgesic duration, and narcotic consumption. Statistical analyses were performed using SPSS v.27 and Plotly.

**Results:**

Subjects exhibited no statistically significant differences in age, gender distribution, duration of surgery, or anesthesia. The hemodynamic status assessment in the recovery room showed no significant differences in SPO_2_, PR, or MAP between the groups. However, remifentanil demonstrated superior effectiveness in reducing pain over 24 hours post TKA surgery compared to Apotel, as evidenced by lower average Visual Analog Scale (VAS) scores (P < 0.001), longer duration without the need for narcotic painkillers (P < 0.001), and lower cumulative opioid analgesic consumption in Group R (P < 0.001).

**Conclusions:**

Remifentanil demonstrates superior pain control in a multimodal pain management approach compared to Apotel, providing sustained pain reduction over 24 hours post-surgery. Moreover, remifentanil offers longer-lasting pain relief and results in lower cumulative narcotic painkiller consumption compared to Apotel.

## 1. Background

Total knee arthroplasty (TKA), commonly referred to as knee replacement surgery, is a surgical procedure designed to alleviate chronic knee pain and restore joint function in individuals with end-stage osteoarthritis of the knee, with additional indications including rheumatoid arthritis, peri-articular fractures, or malignancy. However, the latter often necessitates specialized prostheses (1, 2).

The procedure involves a multidisciplinary team, including primary care physicians, orthopedic surgeons, nurses, and therapists. Comprehensive preoperative evaluation, imaging, and soft tissue management are crucial, and different surgical techniques, such as measured resection and gap balancing, can be employed depending on the implant type and surgeon preference (1, 3, 4).

During TKA, the surgeon removes damaged or diseased parts of the knee joint, including the damaged cartilage and bone, and replaces them with prosthetic components made of metal and plastic. These prosthetic components mimic the natural structure and movement of the knee joint, allowing for improved mobility and pain relief (5, 6).

The TKA procedure involves administering anesthesia, making an incision over the knee joint, reshaping damaged bone, implanting prosthetic components, ensuring proper joint alignment, and closing the incision. Subsequently, patients undergo monitored recovery and essential physical therapy and rehabilitation to regain knee joint function and mobility (1, 2, 7).

Total knee arthroplasty (TKA) is considered a highly successful procedure for relieving chronic knee pain and improving the quality of life for individuals with debilitating knee arthritis. It can significantly enhance a patient's ability to perform everyday activities and enjoy a more active lifestyle. However, it is important for patients to follow postoperative instructions, engage in rehabilitation, and maintain a healthy lifestyle to maximize the long-term benefits of the procedure (8, 9).

Postoperative pain management in TKA is crucial for patient comfort and rehabilitation. Traditionally, opioids have been the primary choice for pain relief, but they come with adverse effects. Multimodal analgesia combines various pain relief methods, such as preemptive analgesia, neuraxial anesthesia, peripheral nerve blockade, patient-controlled analgesia, local infiltration analgesia, and oral medications, to achieve superior pain relief while minimizing opioid-related complications (5, 10, 11).

Preemptive analgesia involves administering medications like COX-2 inhibitors before surgery to prevent pain hypersensitivity. Neuraxial anesthesia and peripheral nerve blockade target pain pathways effectively. Local infiltration analgesia is a low-risk technique where a cocktail of anesthetics and analgesics is injected around the surgical site. Patient-controlled analgesia allows patients to self-administer pain relief as needed, reducing the risk of overmedication. Utilizing these methods in combination enhances pain management, promotes knee recovery, reduces opioid consumption, and improves patient satisfaction (5, 10-13).

Inadequately managed pain can result in diminished patient mobility and hinder the rehabilitation process, potentially prolonging the patient's hospital stay and decreasing overall satisfaction. Moreover, timely control of acute pain following TKA is crucial to mitigate potential postoperative complications, prevent patient dissatisfaction, and reduce the risk of chronic pain syndrome. In fact, inadequate pain control after TKA has been associated with long-term complications, including restricted range of motion and the development of chronic pain syndrome (14, 15).

Effective postoperative pain management is crucial as it can prevent chronic pain that persists 2-3 months after surgery, with incidence rates ranging from 5% to 65%, severely impacting patients' quality of life (16). Utilizing preventive analgesic techniques improves pain control (17), enabling early mobility, reducing complications like DVT and PE, shortening hospital stays, and boosting patient satisfaction (18-20).

Overall, optimal pain control in surgical patients brings multiple benefits, including improved cardiac, respiratory, and gastrointestinal function, reduced thromboembolic risks, decreased chronic post-surgical pain, lower mortality in high-risk patients, enhanced participation in physical therapy, and reduced healthcare costs (21). Various methods, including diverse drugs, administration routes, and medical techniques, are employed for post-surgery pain management. Patient-controlled analgesia (PCA) is widely favored for its efficacy and safety (22). Epidural analgesia, femoral nerve blocks, and oral analgesics are also suitable for total knee arthroplasty (23). Orthopedic patients typically receive oral or intravenous opioids for postoperative pain, but these drugs often trigger adverse effects like nausea, vomiting, pruritus, ileus, and constipation. Higher doses can lead to respiratory depression, hypotension, dizziness, confusion, and delirium (24). A preferred approach combines multiple methods. Multimodal analgesics, such as acetaminophen, nonsteroidal anti-inflammatory drugs (NSAIDs), opioids, ketamine, alpha-2 adrenergic agonists, corticosteroids, gabapentinoids, local anesthesia, epidural anesthesia, peripheral nerve blocks, and cryotherapy, offer diverse options (25).

In TKA, opioids and PCA remain crucial for initial relief from moderate to severe post-surgical pain due to their efficacy. However, they may not be well-tolerated, particularly by the elderly. Lumbar epidural analgesia is another common option but can hinder postoperative mobility and cause complications like epidural hematoma and hypotension (26). Acetaminophen, introduced in 1887 and widely available since the 1950s, is a pain reliever and antipyretic. It lacks peripheral anti-inflammatory effects and does not affect platelet function, making it suitable for surgery anytime. Acetaminophen's analgesic mechanism involves central prostaglandin inhibition through the cyclooxygenase pathway. Studies confirm its ability to reduce postoperative pain, especially in fields like orthopedics. Intravenous acetaminophen, known as Apotel, is commonly used for pain control in operating rooms and inpatient wards (27, 28). Remifentanil, an ultra-short-acting synthetic opioid, allows rapid titration and elimination, with effects fading within 5 to 10 minutes after discontinuation. However, patients receiving remifentanil during surgery may experience postoperative hypotension, bradycardia, secondary hyperalgesia, and increased opioid needs (29-31).

## 2. Objectives

Given the prevalence of knee joint replacement surgery as a common orthopedic procedure, and despite advancements in surgical techniques and anesthesia, many patients continue to experience acute postoperative pain. Therefore, it is imperative for healthcare professionals to employ effective strategies to alleviate these issues. To address this concern, this study aims to assess and compare the efficacy of two medications, Apotel and remifentanil, in managing postoperative pain among patients.

### 2.1. Primary Aims

1. To evaluate and compare the effectiveness of Apotel and remifentanil in pain management, as measured by the Visual Analog Scale (VAS) at various time points: after entering the recovery room and 2, 4, 8, 12, and 24 hours postoperatively and overall.

### 2.2. Secondary Aims

1. To compare the hemodynamic status (MAP, SPO_2_, PR) during the recovery period among patients receiving Apotel and remifentanil.

2. To determine and compare the average duration of analgesia until the first request for analgesia in patients treated with Apotel and remifentanil.

3. To assess and compare the total amount of painkiller (narcotic) consumed by patients receiving Apotel and remifentanil.

## 3. Methods

### 3.1. Patients and Design

This double-blind prospective randomized controlled clinical trial was registered and approved by the Iranian Registry of Clinical Trials (IRCT) under registration number IRCT20220812055663N1. Additionally, it received approval from the institutional ethics committee of Arak University of Medical Sciences on May 29, 2022.

The research was conducted at Amir-al-Momenin and Qods Hospitals in Arak, Iran, spanning from June 2022 to September 2023. Prior to their inclusion in the study, patients or their legal representatives provided written informed consent. It's worth noting that the study meticulously adhered to the Consolidated Standards of Reporting Trials (CONSORT) guidelines, and for further details on this aspect, please refer to the supplementary materials. 

**Figure 1.**
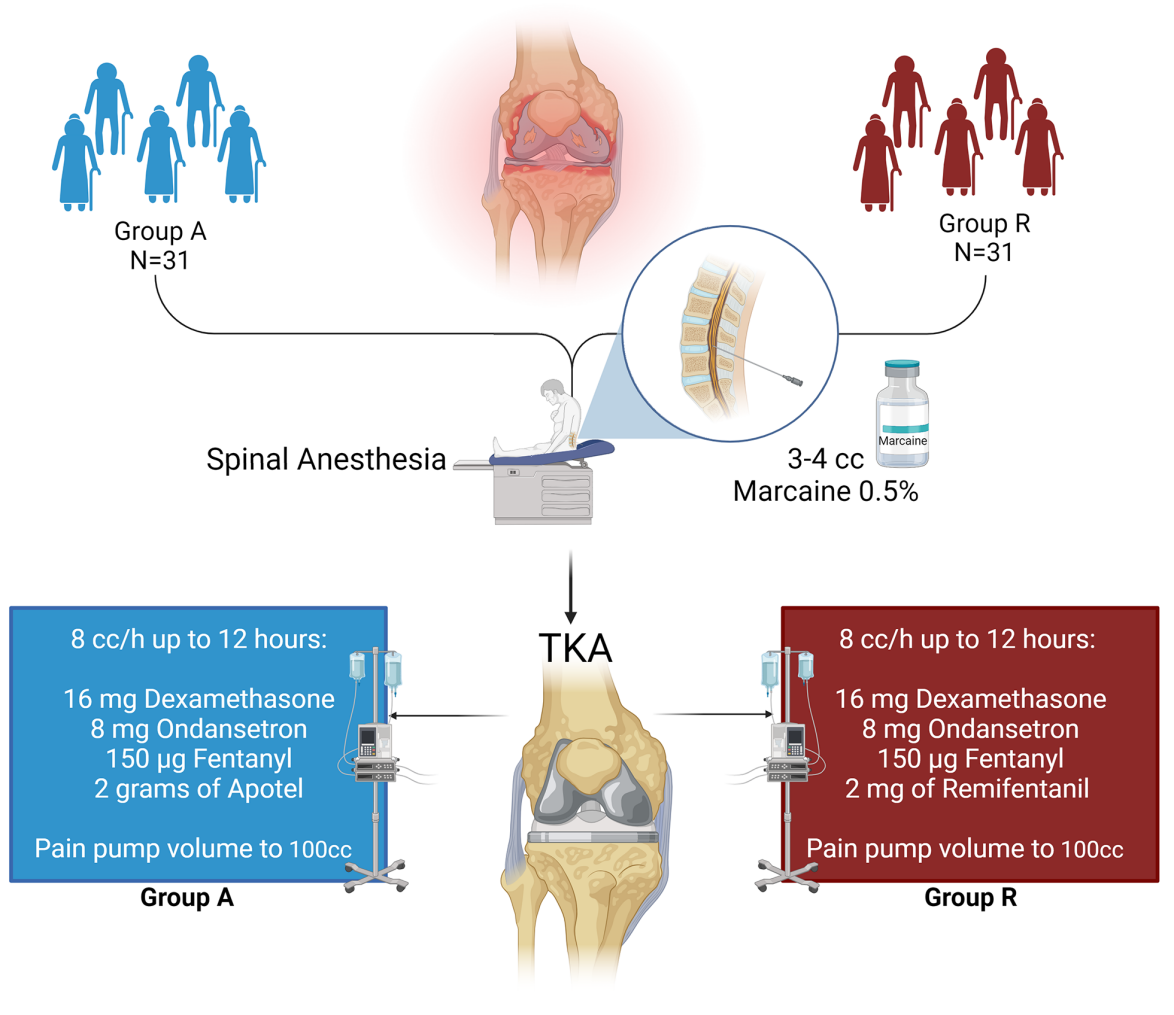
Patients aged 18 - 65 undergoing knee arthroplasty at Amir-al-Momenin and Qods Hospitals were randomly assigned to Group A (Apotel) or Group R (remifentanil) by an anesthesiologist using a randomized block method. Both groups received spinal anesthesia, with Apotel infused in Group A and remifentanil in Group R. Pain management included dexamethasone, ondansetron, fentanyl, and additional post-op drugs in a PCA pain pump based on group allocation.

Within this investigation, a total of 62 eligible patients who were scheduled to undergo knee joint replacement through arthroplasty were randomly assigned to 2 distinct groups: Group A (Apotel) and Group R (remifentanil). Figure 1 presents an overview of the study's design.

#### 3.1.1. Inclusion Criteria

- Eligible candidates for knee joint replacement referred to Amir-al-Momenin and Qods Hospitals, Arak, Iran.

- Age range: 18 to 65 years.

- ASA class I or II.

- Surgery performed by a single qualified surgeon using the same arthroplasty method.

#### 3.1.2. Non-Inclusion Criteria

- Allergies to local anesthetics, remifentanil, Apotel, and opioids.

- Underlying heart, lung, liver, kidney, or other significant medical conditions.

- Patients who have not provided consent to participate.

#### 3.1.3. Exclusion Criteria

- Patients exceeding the maximum surgery duration of 150 minutes.

- Patients experiencing failed spinal anesthesia necessitating a shift to general anesthesia.

### 3.2. Randomization and Blinding

Candidates referred for knee joint replacement, meeting inclusion criteria, were randomly assigned to 2 groups (Apotel and remifentanil) using a randomized block method supervised by an anesthesiologist (A.K.). Initially, the entire series of patients was divided into quadruple blocks (AARR, ARAR, RARA, ARRA, RAAR, RRAA) utilizing computer-generated permuted blocks. Subsequently, randomization within each block was performed to guarantee an equal distribution of patients among the groups.

**Figure 2.**
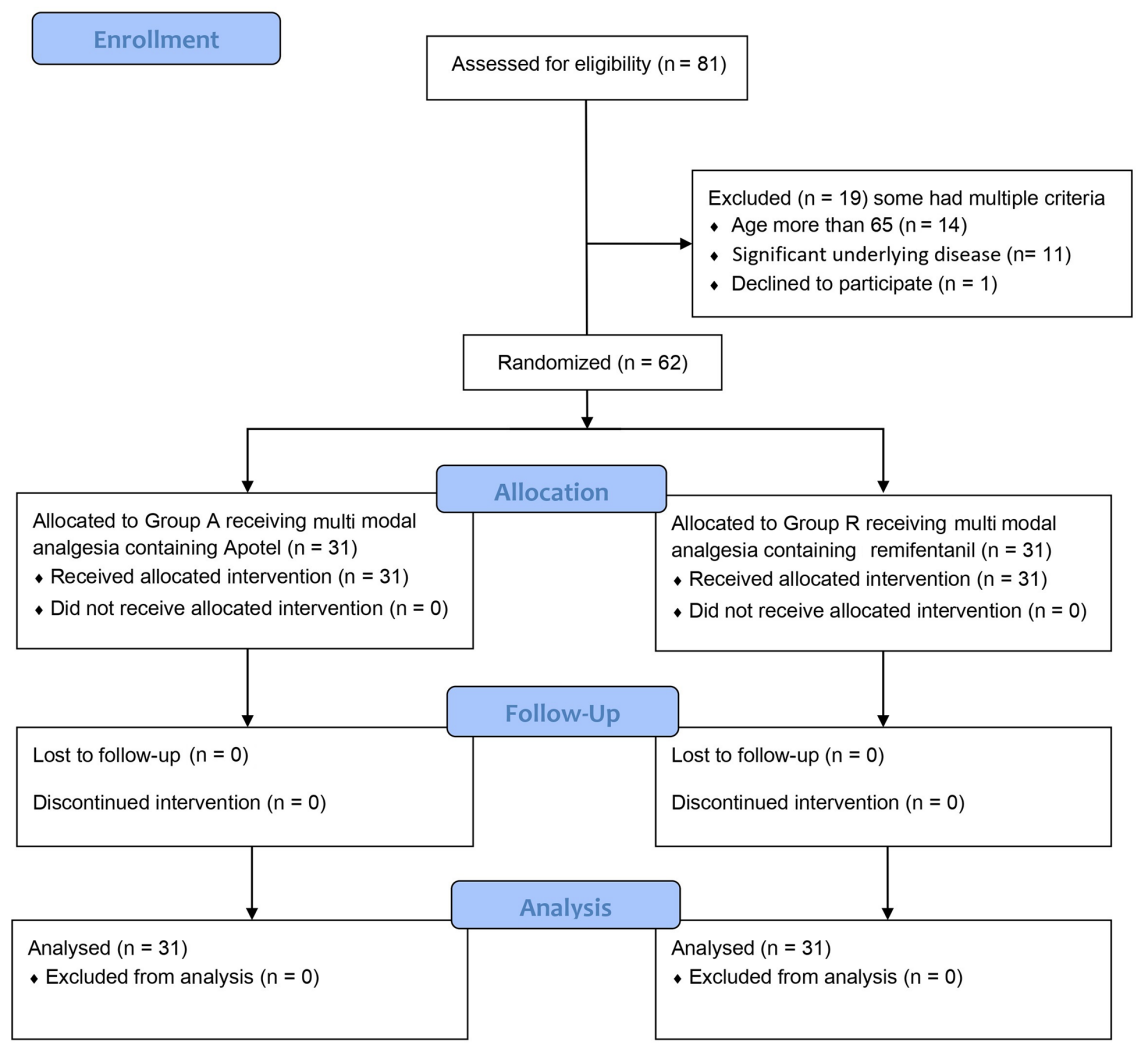
CONSORT Diagram. The flow of the clinical randomized trial is illustrated in a CONSORT diagram. In Group R, remifentanil intervention was administered, while Group A received Apotel.

Following surgery, patients received either remifentanil or Apotel through a pain pump based on their assigned group. To maintain the study's double-blind nature, patients were equally allocated to Group A and Group R, and they received their respective infusions. Importantly, neither the researchers responsible for data collection, clinicians (except the chief anesthesiologist), patients, nor their families were made aware of their group assignments. The project's data collection process remained blind to group distinctions (Figure 2). 

The trial's design, data collection, and analysis were conducted by the authors, who diligently ensured accuracy, completeness, and strict adherence to the protocol. Eligible subjects for TKA, as assessed by the orthopedic surgeon (M.P.), were further evaluated by an anesthesiologist (A.K.) following the study's protocol to determine their eligibility for participation.

### 3.3. Trial Interventions

After obtaining informed consent from the patients, they were brought into the operating room and positioned supine on the operating bed. Upon entering the operating room, all patients underwent comprehensive monitoring of vital signs, including heart rate (HR), respiratory rate (RR), non-invasive blood pressure (NIBP), oxygen saturation (SPO_2_), and temperature. ECG monitoring was initiated, followed by the administration of 3 - 5 cc/kg of crystalloid solution as compensatory volume expansion (CVE). Subsequently, the patients were positioned in a seated posture, and after receiving CVE fluid, a spinal needle (gauge 25) was employed to administer 3 - 4 cc of 0.5% Marcaine through the L4-L5 or L5-S1 space for spinal anesthesia. Following spinal anesthesia, patients were placed in the supine position, and once anesthesia was confirmed, the surgical procedure commenced.

In the Apotel group, following spinal anesthesia and confirmation of hemodynamic stability, 1 gram of Apotel was mixed with 200 cc of normal saline and administered via infusion within the first hour of surgery. In the remifentanil group, after spinal anesthesia, hemodynamic stability was confirmed, and anesthesia was verified; a remifentanil infusion at a rate of 0.5 μg/kg/min was initiated within the first hour of surgery.

Upon completion of the surgical procedure, while ensuring stable hemodynamics and confirming anesthesia, patients were transferred to the recovery room. The anesthesiologist in charge of the plan prepared a pain pump, which was set to administer an 8 cc/h infusion rate for up to 12 hours post-surgery. Both groups received pain management consisting of 16 mg of dexamethasone, 8 mg of ondansetron, 150 µg of fentanyl, and its volume increased to 100 cc using normal saline. For the Apotel group, an additional 2 grams of Apotel were added to the pain pump, while in the remifentanil group, 2 mg of remifentanil (equivalent to 1 vial) were added to the pain pump under the supervision of the project's anesthesiologist. The infusion pump maintained a constant flow of 8 cc/h, and patients had the option for an extra 0.5 cc injection from this mixture every 20 minutes as desired through patient-controlled analgesia (PCA).

It's important to mention that should any patient in either group report a VAS score exceeding 5 during the study, they would receive an IV narcotic injection (pethidine 25 - 50 mg). Furthermore, the quantity of narcotics administered within the 24-hour period following the operation was also carefully documented.

In this study, remifentanil (Remifentanilo, Laboratorios Normon SA, Tres Cantos, Madrid, Spain) and paracetamol (Apotel, Exir Pharmaceutical Co., Tehran, Iran) were utilized.

### 3.4. Measurements

#### 3.4.1. Hemodynamic Monitoring

Throughout the recovery period, continuous monitoring of crucial hemodynamic parameters, including Mean Arterial Pressure (MAP), Peripheral Oxygen Saturation (SPO_2_), and Pulse Rate (PR) were conducted. These vital signs were vigilantly observed and documented using a questionnaire.

#### 3.4.2. Pain Assessment

The Visual Analog Scale (VAS) is a pain assessment tool widely used in clinical settings to measure a person's subjective perception of pain intensity. It consists of a 10-centimeter line, with “no pain" at one end (0 cm) and the “worst pain imaginable" at the other end (10 cm). Patients mark a point on the line that represents their current level of pain, and the distance from the “no pain" end to the marked point is measured in centimeters, providing a quantifiable score for pain intensity. VAS is a straightforward and versatile method for individuals to self-report their pain. In this study, each patient's VAS score was recorded at baseline, upon entering the recovery room, and then at 2, 4, 8, 12, and 24 hours following the operation using a questionnaire (32, 33).

#### 3.4.3. Analgesic Duration Assessment

The average duration of the period from initiation of analgesic intervention to the patient's initial request for a narcotic painkiller injection was recorded and documented using a questionnaire.

#### 3.4.4. Narcotic Consumption

The total amount of narcotic painkillers consumed by each patient was thoughtfully documented in a questionnaire.

Data recording was carried out by S.G., who was kept unaware of the grouping information to ensure the study's masking.

#### 3.4.5. Sample Size Estimation

The sample size for a two-sample test comparing means is determined using a formula based on the normal distribution. This formula incorporates parameters such as standard deviations (δ1 and δ2), means (μ_1_ and μ_2_), and critical values for the desired level of significance (α) and power (1−β). The critical values, $Z_{1-\frac{\alpha }{2}}$ and $Z_{1-\beta }$ represent the thresholds for the two-tailed test at significance level α and the desired power 1−β, respectively. Given specific values for these parameters, the sample size per group (n) is calculated using the formula:


$$n=\frac{{\left(Z_{1-\frac{\alpha }{2}}+Z_{1-\beta }\right)}^{2}\times {\left(\delta_{1}+\delta_{2}\right)}^{2}}{{\left(\mu_{1}-\mu_{2}\right)}^{2}}$$

The mean and standard deviations were extracted from the study by Grape et al. (30). Assuming a significance level (α) of 0.05 and a power of 0.80, the estimated sample size (n) is approximately 31 for each group based on these parameters.

### 3.5. Statistical Analysis

Descriptive statistics were utilized to summarize data characteristics, with categorical variables presented as frequencies and percentages and continuous variables presented as mean ± standard deviation. *t*-tests and chi‑square tests were employed for inferential analyses. Multivariate Analysis of Variance (MANOVA) was utilized to compare 2 or more continuous dependent variables between the 2 intervention groups. Following the MANOVA test and identification of significant differences among the groups, pairwise comparisons were further explored using the Bonferroni post-hoc test. Statistical significance was considered at a P-value below 0.05. The statistical analysis was conducted using IBM SPSS Statistics 27 (IBM Corp., USA). Additionally, Plotly, a Python open-source library, was utilized to create data visualizations.

## 4. Results

In the final analysis, 62 patients participated in this trial, with an average age of 60.71 ± 3.02 years. The mean age in Group A was 60.65 ± 2.96, while in Group R, it was 60.77 ± 3.13. Notably, there was no statistically significant difference in age between the 2 groups (P = 0.868). Among the participants, 24 (77.4%) in Group A were female, and 21 (67.7%) in Group R were also female, with gender distribution showing no significant distinction between the intervention groups (P = 0.393). There was no statistically significant difference in the duration of surgery (P = 0.602) or anesthesia (P = 0.737) between the two groups. Detailed demographic and surgical data are presented in Table 1. 

**Table 1.** Demographics and Surgical Data ^a^

Demographics	Group A (N = 31)	Group R (N = 31)	P-Value
**Mean age, y**	60.65 ± 2.96	60.77 ± 3.13	0.868
**Gender**			
Male	7 (22.6)	10 (32.3)	0.393
Female	24 (77.4)	21 (67.7)	
**Surgery time, min**	119.6 ± 11.6	121.2 ± 12.4	0.602
**Anesthesia time, min**	178.4 ± 15.4	179.7 ± 14.9	0.737
**Blood loss, mL**	355 ± 135	372 ± 131	0.617

^a^ The data are depicted as the mean ± SD or No. (%). Group R underwent remifentanil intervention, whereas Group A was administered Apotel. The statistical analysis comprised a chi-squared test for gender and an independent samples *t*-test for age comparisons between the two groups.

In the recovery room after surgery, we assessed the participants' hemodynamic status, including measurements of SPO_2_, PR, and MAP. The average SPO_2_ in Group A was 98.87 ± 0.81, while in Group R, it was 98.94 ± 0.89, and we found no significant difference in SPO_2_ between the two groups (P = 0.766). Likewise, the mean PR in Group A was 69.35 ± 4.29, and in Group R, it was 68.81 ± 3.87, with no significant difference observed between the groups (P = 0.599). Additionally, the mean MAP in Group A was 86.10 ± 1.89, and in Group R, it was 85.67 ± 2.78, again showing no significant difference in MAP between the groups (P = 0.475). Detailed hemodynamic data are presented in Table 2. 

**Table 2.** Effects of Apotel and Remifentanil on Hemodynamic Status ^a^

Variables	Group A (N = 31)	Group R(N = 31)	P-Value
**MAP**	86.10 ± 1.89	85.67 ± 2.78	0.475
**PR**	69.35 ± 4.29	68.81 ± 3.87	0.599
**SPO**_**2**_	98.87 ± 0.81	98.94 ± 0.89	0.766

Abbreviations: SPO_2_, peripheral oxygen saturation; MAP, mean arterial pressure; PR, pulse rate.

^a^ The data are presented as mean ± SD and were subjected to a *t*-test for analysis. In Group R, remifentanil intervention was administered, while Group A received Apotel.

To assess the impact of the intervention group (Apotel vs. remifentanil) on patients' average VAS pain scores at six-time points (0, 2, 4, 8, 12, and 24 hours after surgery), we conducted a Multivariate Analysis of Variance (MANOVA). The intervention group served as the independent variable, with 6 VAS scores as dependent variables. The MANOVA results indicated a significant overall effect of the intervention group on patients' average VAS scores (F = 14.12, P < 0.001). Additionally, Wilk's Λ demonstrated a notable between-group difference (Λ = 0.393), indicating distinct effects of the 2 intervention groups on the set of 6 VAS variables collectively. The substantial effect size (partial η^2^ = 0.607) underscores the clinical significance of these observed differences.

**Figure 3.**
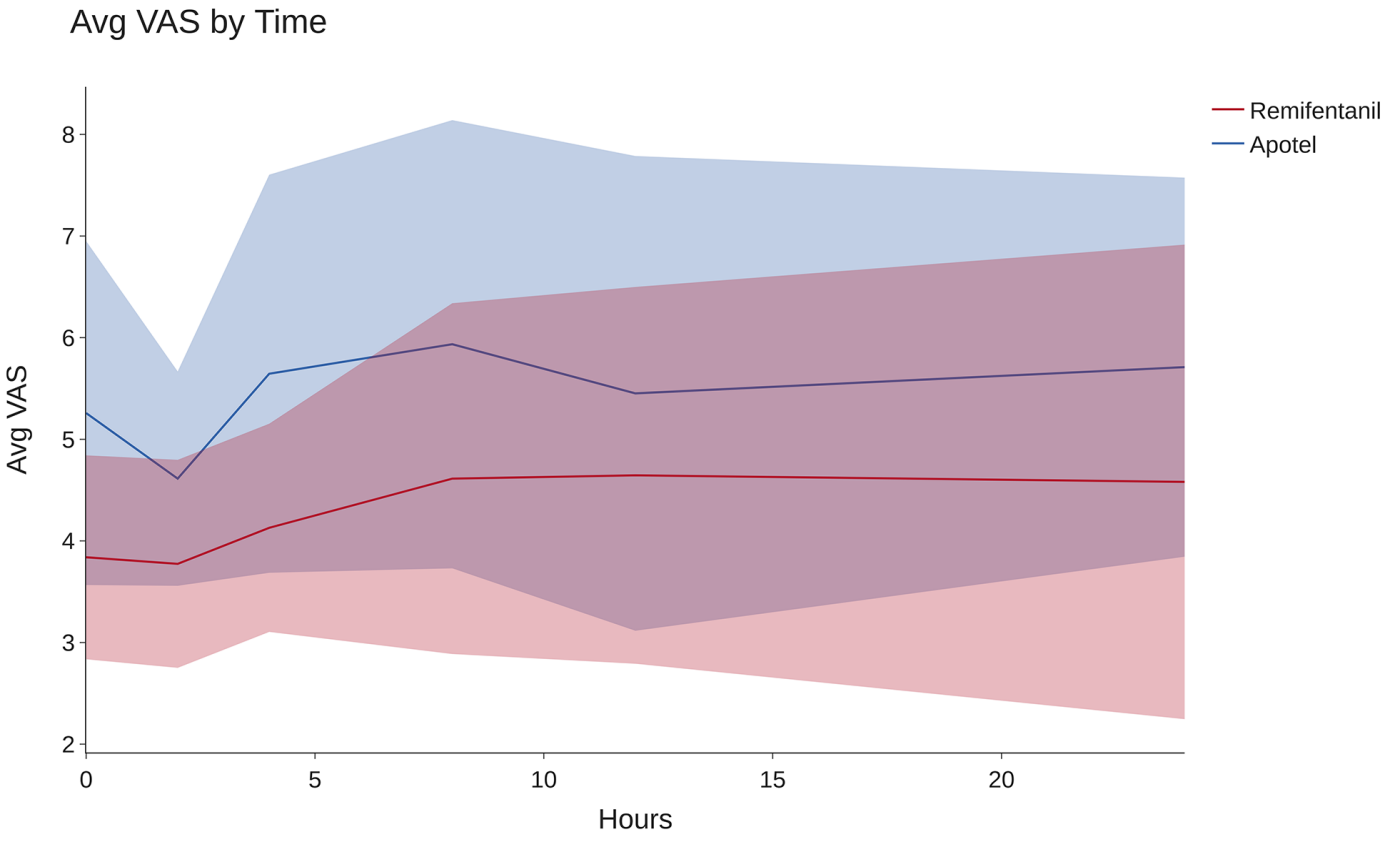
Average VAS by Group. This figure illustrates the changes in average VAS scores within the initial 24 hours following TKA surgery for each group. The lines represent the mean values, and the shaded areas represent the standard deviations.

As depicted in Figure 3, remifentanil is significantly more effective in reducing pain 24 hours post-TKA surgery (P < 0.001). To assess the impact of the interventions on each dependent variable individually, pairwise comparisons between groups were conducted using the Bonferroni post hoc test, with a Bonferroni-adjusted alpha level of 0.025, as outlined in Table 3. 

**Table 3.** VAS vs. Group Pairwise Comparisons ^a^

Dependent Variables	Mean± SE		P-Value
	Group A	Group R	
**VAS (recovery)**	5.258 ± 0.250	3.839 ± 0.250	< 0.001
**VAS (2 hrs-PO)**	4.613 ± 0.187	3.774 ± 0.187	0.002
**VAS (4 hrs-PO)**	5.645 ± 0.281	4.129 ± 0.281	< 0.001
**VAS (8 hrs-PO)**	5.935 ± 0.356	4.613 ± 0.356	0.011
**VAS (12 hrs-PO)**	5.452 ± 0.379	4.645 ± 0.379	0.137
**VAS (24 hrs-PO)**	5.710 ± 0.380	4.581 ± .380	0.040

^a^ Bonferroni post-hoc tests were conducted for pairwise comparisons within the MANOVA analysis. The data are expressed as the mean ± standard error (SE). Group R underwent remifentanil intervention, whereas Group A was treated with Apotel. VAS refers to the Visual Analog Scale, and PO signifies the post-operative period.

The post hoc results revealed that the average VAS scores at baseline, 2 hours, 4 hours, 8 hours, and 24 hours following the intervention were significantly lower in the remifentanil group (all P-values < 0.05). However, there was no statistically significant difference in the average VAS scores between the groups at the 12-hour post-op time point (P = 0.130).

To evaluate the duration of pain relief provided by the interventions, we measured the time until the first request for a narcotic painkiller following surgery. In Group A, the average analgesic duration was 267.10 ± 249.241 minutes, whereas in Group R, it extended to 855.48 ± 408.11 minutes. This substantial difference indicates that Group R experiences significantly longer periods without the need for narcotic painkiller consumption compared to Group A (P < 0.001). See Table 4. 

**Table 4.** Effects of Apotel and Remifentanil on Opioid Consumption After Surgery and Time to First Request for Analgesic ^a^

Variables	Group A (N = 31)	Group R (N = 31)	P-Value
**Analgesic time, min**	267.10 ± 249.24	855.48 ± 408.11	< 0.001
**Analgesic demand (opioid) mg**	8.71 ± 3.64	4.68 ± 2.87	< 0.001

^a^ The data are presented as mean ± SD and were subjected to a *t*-test for analysis. In Group R, remifentanil intervention was administered, while Group A received Apotel.

The cumulative consumption of narcotic painkillers amounted to 8.71 ± 3.64 mg in Group A and 4.68 ± 2.87 mg in Group R, illustrating a notably greater demand for opioid analgesics in Group A (P < 0.001). See Table 4. 

## 5. Discussion

Total knee arthroplasty is a well-established surgical procedure for individuals suffering from debilitating knee arthritis. It significantly improves patients' quality of life and mobility, but postoperative pain management is crucial for successful recovery. Traditional opioid-based pain relief methods are effective but often come with adverse effects, necessitating the exploration of alternative approaches like multimodal analgesia.

In this study, 62 eligible patients with an average age of 60.71 ± 3.02 years underwent TKA and were randomly assigned to two groups: Apotel (Group A) and remifentanil (Group R). After undergoing spinal anesthesia and surgery, patients in each group received either a remifentanil- or Apotel-based pain pump for postoperative pain management. The study aimed to compare the effectiveness of Apotel and remifentanil in managing postoperative pain after TKA, with hemodynamic measurements showing no significant differences between the groups. Remifentanil demonstrated superior pain control over a 24-hour period, as indicated by lower VAS scores at various time points. Additionally, Group R experienced longer-lasting pain relief and lower cumulative consumption of narcotic painkillers compared to Group A.

In their 2017 clinical trial, O'Neal et al. randomly assigned 174 patients into three groups: one receiving intravenous acetaminophen, another receiving oral acetaminophen (both at a dose of 1 gram), and a third receiving an oral placebo. The study demonstrated that when used as an adjunct pain relief medication alongside hydromorphone, there were no significant differences in pain intensity among patients during the first 6 hours and the first 24 hours after surgery between the oral and injectable acetaminophen groups. These findings align with our study, indicating that the utilization of acetaminophen (Apotel) does not significantly impact average pain levels during the initial 24 hours following surgery (34).

In a single-blinded, randomized clinical trial at Taleghani Hospital in Iran, 70 patients undergoing elective C-sections were randomly assigned to receive either intravenous apotel or remifentanil. The study found that remifentanil provided superior postoperative pain control compared to apotel immediately after surgery, as indicated by lower pain scores during the recovery period. However, there were no significant differences between the groups in terms of narcotic drug use, blood pressure, or heart rate (35). These findings align with our study results, suggesting that remifentanil's superior postoperative pain reduction performance may be extended to various other surgical procedures (35).

A study conducted in Korea to assess the impact of intraoperative remifentanil infusion on postoperative opioid consumption in patients undergoing TKA with femoral nerve block aimed to investigate whether the use of remifentanil during surgery would influence the amount of opioids required for postoperative pain control. It showed that patients who received intraoperative remifentanil had a significantly higher cumulative opioid consumption at 48 hours postoperatively compared to those who did not. This finding suggests a potential link between remifentanil use and opioid-induced hyperalgesia, leading to increased postoperative pain and opioid requirements. Additionally, the study highlighted the importance of multimodal pain management strategies and the need to balance effective pain control with minimizing opioid-related side effects (13).

This study contradicts our findings, as our research showed that intraoperative remifentanil reduces postoperative opioid consumption. This inconsistency likely arises from the discontinuation of postoperative remifentanil infusion and the use of tramadol-fentanyl pumps in this study. When combining our results with those of this study, it becomes evident that relying solely on intraoperative remifentanil administration, without postoperative remifentanil-based pain pumps, could potentially lead to an increased demand for opioid analgesics and is not advisable. If remifentanil is planned for TKA analgesia, it should also be factored into postoperative pain management considerations, as it may contribute to opioid-induced hyperalgesia (13).

The study by Tomita et al. investigated acute opioid tolerance during remifentanil infusion for postoperative pain in patients undergoing TKA. Findings suggest that intraoperative remifentanil infusion led to increased postoperative pain during movement, indicating potential acute tolerance development, while preoperative NSAID administration showed some promise in improving postoperative analgesia (36). These findings suggest that while restricting the use of remifentanil during surgery may elevate the risk of opioid tolerance and lead to less favorable outcomes, its continued administration in the postoperative phase has the potential to reduce the need for opioid analgesics.

The study conducted by Hwang et al. investigated the use of dexmedetomidine as an adjuvant in propofol-based total intravenous anesthesia (TIVA) for spinal surgery. Dexmedetomidine displayed superior efficacy to remifentanil in controlling postoperative pain for up to 48 hours after surgery, reducing the need for rescue analgesics and postoperative nausea and vomiting (PONV), potentially making it a more efficient choice in propofol-based TIVA for pain and PONV management. It is important to note that in this study, dexmedetomidine and remifentanil were exclusively used for intraoperative pain management, with patient-controlled pumps containing tramadol and fentanyl for postoperative pain control (37).

A pilot study compared two intravenous patient-controlled analgesia regimens with different doses of remifentanil for labor analgesia. Although pain and satisfaction scores were similar in both groups, the regimen with a continuous infusion of 0.025 - 0.1 microg per kg per min and a bolus of 0.25 microg per kg in Group A was associated with fewer side effects compared to the bolus-based dosing regimen in Group B, suggesting the potential efficacy of remifentanil intravenous PCA for labor analgesia, but close respiratory monitoring is necessary due to the potential for respiratory depression (38).

A review of 20 randomized controlled trials involving 3,569 women compared the use of remifentanil intravenous PCA with various other analgesic methods for labor pain relief. The review found that women using remifentanil PCA reported higher satisfaction with pain relief and stronger pain relief at one hour compared to some other opioids but also noted that remifentanil PCA was associated with increased pain intensity compared to epidural analgesia. However, the quality of evidence was generally low, and further research is needed to assess the safety and efficacy of remifentanil PCA for both mothers and newborns during labor (39).

Remifentanil postoperative infusion has demonstrated superiority in post-operative pain control, offering sustained relief over 24 hours and reducing cumulative narcotic consumption. Although concerns arise regarding potential opioid-induced hyperalgesia, acute tolerance development, and the need for careful patient selection, these concerns are related to limited intraoperative use, and postoperative use may reduce or mask this effect. In certain surgical contexts, such as spinal surgery, dexmedetomidine may offer better pain management with fewer side effects than remifentanil, prompting the need for a nuanced approach to opioid usage in postoperative care. Additionally, remifentanil's application in labor analgesia yields high patient satisfaction but necessitates vigilant respiratory monitoring due to the risk of respiratory depression. Hence, the choice of remifentanil for postoperative pain management should be considered in light of specific surgical requirements and the potential for opioid-related complications, emphasizing the importance of a comprehensive, multimodal pain management strategy and the need for further research to determine its appropriateness and safety in various clinical scenarios.

Remifentanil has demonstrated its effectiveness in post-operative pain control, offering sustained relief over a 24-hour period and reducing cumulative narcotic consumption. However, concerns have been raised regarding potential issues such as opioid-induced hyperalgesia and acute tolerance development. It's important to note that these concerns are often associated with limited intraoperative use, and postoperative use of remifentanil may reduce or mask these effects.

In specific surgical contexts, such as spinal surgery, dexmedetomidine has shown promise as a potential alternative to remifentanil, providing better pain management with fewer side effects. This highlights the importance of adopting a nuanced approach to opioid usage in postoperative care, considering the unique requirements of each surgical procedure.

Moreover, when considering remifentanil in other contexts, such as labor analgesia, it's worth noting that it can yield high patient satisfaction. However, this approach necessitates vigilant respiratory monitoring due to the potential risk of respiratory depression.

In conclusion, the choice of using remifentanil for post-operative pain management should be carefully evaluated in light of specific surgical requirements and the potential for opioid-related complications. Emphasizing the importance of a comprehensive, multimodal pain management strategy is crucial, and further research is needed to determine the appropriateness and safety of remifentanil in various clinical scenarios.

### 5.1. Limitations

This study, conducted with a small sample size of 62 patients in Arak, Iran, may limit the generalizability of its findings to broader healthcare contexts. The study primarily assessed pain control within the first 24 hours after surgery. It is crucial for future studies to investigate pain control for extended periods to ensure that postoperative remifentanil will mitigate analgesic tolerance or merely mask it for a short period of time. The study did not consider potential confounding factors like baseline pain levels, psychological variables, or concurrent medications. Therefore, while it offers valuable insights into the effectiveness of Apotel and remifentanil in postoperative pain management after total knee arthroplasty, its limitations, including the small sample size, absence of a placebo group, and potential confounding factors, should be noted when interpreting the results.

### 5.2. Conclusions

This study compared Apotel and remifentanil for postoperative pain management in TKA patients, revealing several key findings. The research included 62 participants with no significant differences in demographics or surgical characteristics between the 2 intervention groups. Remifentanil exhibited superior and sustained pain control over 24 hours, with longer-lasting pain relief and lower cumulative narcotic painkiller consumption compared to Apotel. Hemodynamic parameters remained stable for both medications.

## Data Availability

The dataset presented in the study is available on request from the corresponding author during submission or after publication.

## Clinical Trial Registration Code:

The research, which is a double-blind prospective randomized controlled clinical trial, has been duly registered and approved by the Iranian Registry of Clinical Trials (IRCT) under the registration number IRCT20220812055663N1.
